# Growth impairment in glycogen storage disease type I versus types III/VI/IX: a cross-sectional study

**DOI:** 10.1186/s12887-025-06053-1

**Published:** 2025-10-06

**Authors:** Xiaohui Wu, Yueyu Sun, Min Yang

**Affiliations:** Department of Pediatrics, Guangdong Provincial People’s Hospital, Guangdong Academy of Medical Sciences, Southern Medical University, Guangzhou, 510080 China

**Keywords:** Glycogen storage disease (GSD), Growth Impairment, IGF1 (Insulin-like Growth Factor 1), Pediatric EndocrinologyMetabolic DisordersRare Genetic Disorders

## Abstract

**Background:**

Growth retardation is common in glycogen storage disease (GSD), though the relative contributions of hormonal and metabolic factors remain unclear. We compared clinical and biochemical features between GSD I and non-GSD I patients and identified independent predictors of height standard deviation score (SDS).

**Methods:**

Thirty-eight children with GSD (24 with GSD I; 14 with GSD III/VI/IX; mean age: 7.5 years) underwent evaluation of height SDS, BMI SDS, IGF1 SDS, and metabolic parameters. After excluding three patients with inflammatory bowel disease (final *n* = 35), multiple regression was used to identify factors associated with height SDS. In GSD I (*n* = 24), Lasso regression selected variables, and 1,000 bootstrap resamples assessed coefficient stability.

**Results:**

GSD I patients had lower height SDS (–2.30 vs. − 1.17; *p* = 0.021) and higher lactate (3.94 vs. 1.48 mmol/L; *p* < 0.001), uric acid (431.04 vs. 283.79µmol/L; *p* < 0.001) and triglyceride levels (2.38 vs. 1.29 mmol/L, *p* = 0.002) compared to non-GSD I. In combined-cohort regression, lactate was the only independent negative predictor of height SDS (*p* = 0.011); glucose levels and IGF1 SDS did not reach statistical significance. In GSD I, Lasso retained lactate (β = − 0.682), glucose (β = − 0.625), and IGF1 SDS (β = 0.524), and bootstrap validation showed only IGF1 SDS remained consistently significant.

**Conclusions:**

Hyperlactatemia is significant predictor of growth impairment in GSD, while IGF1 is a stable predictor in GSD I. These findings highlight metabolic and hormonal targets for future hypothesis-driven research in this population.

**Supplementary Information:**

The online version contains supplementary material available at 10.1186/s12887-025-06053-1.

## Background

Glycogen Storage Diseases (GSDs) are a group of rare inherited metabolic disorders caused by deficiencies in enzymes or transporters responsible for glycogen metabolism [1]. These deficiencies result in the abnormal accumulation of glycogen in various tissues, primarily the liver, muscles, and kidneys, leading to metabolic disturbances such as hypoglycemia, hepatomegaly, and muscle weakness [2]. Among hepatic GSDs, Type I (GSD I) is the most extensively studied, owing to its severe impairment of both glycogenolysis and gluconeogenesis, which provokes profound fasting hypoglycemia, lactic acidosis and marked growth retardation [3, 4]. In contrast, GSD Type III (GSD III), Type VI (GSD VI) and Type IX (GSD IX) preserve intact gluconeogenic pathways; patients therefore tend to demonstrate milder fasting hypoglycemia, but may manifest with postprandial hyperglycemia and hyperlactacidemia [5, 6].

Growth impairment is a common clinical feature in patients with GSD, particularly in childhood [7, 8]. Affected individuals often present with short stature, and the severity of growth delay varies by the specific GSD subtype, age at diagnosis and quality of metabolic control [9, 10]. Previous observational studies have reported associations between metabolic factors, nutritional status, and hormonal regulation with growth outcomes in GSD, but the precise nature of these relationships remains unclear.

Among the factors linked to growth regulation, insulin-like growth factor 1 (IGF1) is a key mediator of growth hormone activity and an important determinant of linear growth. In GSD patients, both nutritional status and liver function—which strongly influence circulating IGF1 levels—are often compromised. Some reports have described lower IGF1 concentrations in GSD cohorts, yet the degree to which IGF1 correlates with height or growth velocity in these patients has not been definitively established [11, 12].Nutritional status—commonly assessed via body mass index (BMI)—has likewise been examined as a possible correlate of growth, but data on its relationship with stature in GSD remain limited.

Metabolic disturbances in glucose and lactate handling have also been evaluated for their potential links to growth outcomes [13, 14]. For example, recurrent hypoglycemia and lactic acidosis in GSD I result from impaired glycogenolysis and gluconeogenesis, and elevated lactate levels have been observed alongside various metabolic derangements. However, how these biochemical parameters co-vary with measures of growth has not been fully characterized.

This study therefore aims to investigate the hormonal and metabolic factors associated with growth in GSD patients—specifically, to assess the correlations between height (or height SDS) and IGF1 levels, BMI, and key metabolic parameters. By focusing on these observational associations, we hope to clarify which clinical and biochemical measures co-occur with growth outcomes in GSD, thereby informing future hypothesis-driven research and potential strategies for optimizing growth in this population.

## Methods

### Study design and patient selection

This cross-sectional investigation was conducted at Guangdong Provincial People’s Hospital. We included pediatric patients (*n* = 38) with a confirmed diagnosis of hepatic glycogen storage disease (GSD types I, III, VI, and IX) who had received regular followup care and treatment from a pediatric gastroenterologist or medical geneticist for at least 12 months. Confirmation of GSD diagnosis was based on clinical presentation, biochemical testing, and genetic analysis. When available, molecular data—defined as pathogenic or likely pathogenic variants in GSD‐associated genes—were extracted from medical records. Table S1 summarizes variants identified in 22 patients; remaining cases met clinical and biochemical criteria unequivocally and are denoted “N/A” for genetic results. Due to the study’s crosssectional nature, initial diagnoses at external centers, and long intervals to followup, comprehensive genetic reports were not uniformly retrievable. However, each patient had documentation of at least pathogenic or likely pathogenic variant in the pertinent gene.

Exclusion criteria comprised the presence of other metabolic or endocrine disorders known to affect growth, as well as incomplete clinical or biochemical data. Enrolled patients were stratified into two cohorts: GSD I (*n* = 24) and non–GSD I (types III, VI, or IX; *n* = 14). The demographic profile included both male and female participants, with a mean age of 7.5 years (range, 2.2–14.0 years).

### Data collection

All assessments were performed at the most recent routine follow-up visit, scheduled immediately prior to the patients’ subsequent uncooked cornstarch dose.

Anthropometric Measurements: Height and weight were measured according to standard pediatric protocols, with patients barefoot and in light clothing. Both measurements were taken in duplicate and averaged. Height was measured to the nearest 0.1 cm, and weight to the nearest 0.1 kg. All measurements were performed by Dr. Yueyu Sun, a trained pediatric endocrinologist. Height SDS were calculated against the Chinese national growth reference for children and adolescents aged 0–18 years, with short stature defined as height < − 2 SDS for age and sex [15]. BMI was calculated as weight (kg) divided by height squared (m²) and converted to BMI SDS based on pediatric reference standards.

Serum IGF1 Levels: Measured using an immunoassay (chemiluminescent immunoassay). IGF1 values were adjusted for age and sex according to established pediatric reference ranges.

Biochemical Markers: Metabolic parameters, including lactate, glucose, uric acid (UA), triglycerides (TG), alanine aminotransferase (ALT), aspartate aminotransferase (AST), and total cholesterol (TC), were analyzed. Lactate was measured using enzymatic assays, with normal reference values ranging from 0.60 to 2.20 mmol/L. Blood glucose levels were obtained from venous samples, with normal fasting glucose levels ranging from 3.9 to 6.1 mmol/L. UA, TG, ALT, AST, and TC levels were measured using standard biochemical methods.

Clinical Comorbidities: Information regarding hepatic and renal complications or any additional comorbidities affecting growth was documented.

### Statistical analysis

Continuous variables are presented as either mean ± SD or median (IQR) for non-normal distributions, and categorical data are presented as counts (%). Baseline comparisons between the GSD I and non–GSD I groups were performed using Student’s t-test (or Mann–Whitney U test when appropriate) for continuous variables, and χ² or Fisher’s exact test for categorical variables.

To identify independent predictors of linear growth in the combined cohort (*n* = 35), multiple linear regression was conducted with height SDS as the dependent variable, and IGF1 SDS, BMI SDS, lactate, and glucose as independent variables. Model assumptions—including linearity, homoscedasticity, normality of residuals, and the absence of multicollinearity (all VIF < 2)—were tested. Statistical significance was set at a two-sided *p* < 0.05. Analyses were performed using SPSS version 27.0 (SPSS Inc., Chicago, IL, USA). Additional analysis was conducted in R (version 4.4.1) using the glmnet package (Friedman, Hastie, and Tibshirani, 2010). Predictor variables were centered and scaled prior to modeling. Within the GSD I cohort (*n* = 24), we fitted a LASSO regression model (α = 1) to identify independent predictors of height SDS. The optimal penalty parameter, λ, was selected via 10-fold cross-validation (cv.glmnet()), choosing the value of λ (lambda.min) that minimized the mean cross-validated error. To evaluate the stability of the estimated coefficients, we conducted 1,000 bootstrap resamples: for each resample, we drew a sample of the same size as the original dataset with replacement, refitted the Lasso model, and recorded the distribution of each coefficient to calculate 95% confidence intervals.

## Results

### Patient characteristics

A total of 38 patients were included in this study, comprising 24 patients diagnosed with GSD I and 14 patients diagnosed with other GSD types (GSD III, VI, IX). Within the non-GSD I group, two patients were classified as GSD III (one GSD IIIa and one GSD IIIb). Because a large international GSD registry has demonstrated no significant difference in growth patterns between GSD IIIa and GSD IIIb [16], we combined these two individuals into a single GSD III subgroup for all subsequent analyses.

The mean age of the cohort was 7.5 years (range: 2.2–14 years). The majority of patients were diagnosed in infancy. All of them receive dietary management and use uncooked cornstarch. No patients in the study cohort exhibited liver adenomas or renal dysfunction at the time of data collection. One GSD Ia patient was diagnosed as colitis and two GSD Ib patients were diagnosed as Crohn’s disease. The demographic and clinical characteristics of the patients are summarized in Table 1.

**Table 1:** Baseline characteristics of GSD I and other GSD types patients

Characteristics	GSD I	non-GSD I
n	24	14
Age, mean ± sd	8.5 ± 3.5	5.9 ± 2.7
Gender, n (%)		
Male	16 (42.1%)	12 (31.6%)
Female	8 (21.1%)	2 (5.3%)
GSD type, n (%)		
Ia	13 (34.2%)	0 (0%)
Ib	11 (28.9%)	0 (0%)
9	0 (0%)	6 (15.8%)
6	0 (0%)	6 (15.8%)
3	0 (0%)	2 (5.3%)

This table presents the baseline demographic and clinical characteristics of patients with Glycogen Storage Disease Type I (GSD I) and other GSD types (GSD III, VI, IX). Variables include age, sex distribution

### Growth parameters

The analysis of growth parameters revealed significant differences between GSD I and other GSD types. GSD I patients exhibited significantly lower height-for-age standard deviation scores (height SDS) compared to GSD III, VI, IX patients, indicating more severe growth impairment. The mean height SDS for GSD I patients was − 2.30, whereas the mean height SDS for other GSD types was − 1.17 (*p* = 0.021). Among GSD I patients, 37.5% were classified as having short stature (height SDS < −2), compared to 0% in the other GSD types group.

BMI SDS analysis revealed no significant difference between GSD I and other GSD types (*p* = 0.88). The mean BMI SDS for GSD I patients was 0.63, while for other GSD types, it was 0.71. IGF1 SDS levels were lower in GSD I patients (−1.48) compared to GSD III, VI, IX patients (−1.02), but this difference was not statistically significant (*p* = 0.131).

### Metabolic parameters

A series of metabolic parameters were evaluated for their relationship with growth outcomes: The complete statistical results are summarized in Table 2.

**Table 2:** Growth and metabolic parameters in GSD patients by GSD type

variables	GSD I (*N* = 24)	non-GSD I (*N* = 14)	*p*
Igf1 SDS	−1.48 ± 1.01	−1.02 ± 0.63	0.131
height SDS	−2.3 ± 1.59	−1.17 ± 0.9	0.021
BMI SDS	0.63 ± 1.46	0.71 ± 1.22	0.88
LAC mmol/L	3.94 ± 1.7	1.48 ± 0.47	0.000
ALT U/L	36.13 ± 39.68	83.79 ± 106.99	0.129
AST U/L	52.92 ± 58.68	77.64 ± 69.62	0.25
UA umol/L	431.04 ± 160.55	283.79 ± 55.33	0.000
GLU mmol/L	3.77 ± 1.05	3.46 ± 0.73	0.339
TC mmol/L	4.92 ± 1.31	5.04 ± 0.9	0.76
TG mmol/L	2.38 ± 1.44	1.29 ± 0.53	0.002

This table presents the results of independent sample t-tests comparing various metabolic and growth parameters between patients with Glycogen Storage Disease Type I (GSD I) and other GSD types (GSD III, IV, IX)

Glucose Levels: The mean glucose level in GSD I patients was 3.8 mmol/L, slightly higher than the 3.5 mmol/L observed in GSD III, VI, IX patients, but the difference was not statistically significant (*p* = 0.339).

Lactate Levels: GSD I patients exhibited significantly higher lactate levels compared to other GSD types (3.94 vs. 1.48 mmol/L, *p* < 0.001). Higher lactate levels were negatively correlated with height SDS. Uric Acid (UA) Levels: The mean uric acid level in GSD I patients was significantly higher (431.04 µmol/L) compared to other GSD types (283.79 µmol/L), with a highly significant difference (*p* < 0.001). Triglyceride (TG) Levels: Triglyceride levels were significantly elevated in GSD I patients (mean = 2.38 mmol/L) compared to other GSD types (mean = 1.29 mmol/L), with a significant difference (*p* = 0.002).

Other Biochemical Markers: No significant differences were observed between GSD I and other GSD types for ALT (*p* = 0.129), AST (*p* = 0.250), and total cholesterol (TC) levels (*p* = 0.760).

### Regression analysis

Prior to analysis, three patients with comorbid gastrointestinal conditions (one GSD Ia patient with colitis and two GSD Ib patients with Crohn’s disease) were excluded to minimize confounding effects on growth outcomes. The regression models therefore focus on metabolic and hormonal predictors among the remaining pediatric GSD cohort. The results are detailed below:

Lactate levels was found to have a statistically significant negative association with height SDS with a coefficient of −0.411 (*p* = 0.011), and a standardized Beta of −0.520.

IGF1 SDS showed a marginally non-significant positive relationship with height SDS (B = 0.441, *p* = 0.104), with a standardized Beta of 0.272, suggesting a moderate positive association.

BMI SDS did not have a significant effect on height SDS (B = −0.043, *p* = 0.806).

Glucose levels showed a negative relationship with height SDS (B = −0.439, *p* = 0.092), which approached significance, but did not meet the conventional threshold.

Uric acid and triglycerides were not significantly associated with height SDS (*p* = 0.778 and *p* = 0.745, respectively).

- **Subgroup Analysis (GSD I only**,***n= 24***): Due to the relatively small sample size and the interrelated nature of lactate, glucose, and IGF1 SDS, we applied Lasso regression to perform variable selection while mitigating multicollinearity. The final model retained the following predictors: intercept (–2.3004), lactate (–0.6819), IGF1 SDS (0.5242), and glucose (–0.6246).
- **Bootstrap Validation (1**,**000 resamples)**: Coefficient stability was assessed using bootstrap resampling. Only the IGF1 SDS coefficient remained statistically robust, with its 95% confidence interval not including zero. The bootstrap 95% confidence intervals for lactate and glucose both crossed zero, indicating that their effects were less consistent under resampling variability.

These findings suggest that, within the GSD I subgroup, IGF1 SDS is the most reliable predictor of height SDS when accounting for metabolic variables. While lactate and glucose may still contribute to growth variation, their effects appear more sensitive to sampling variability than IGF1 SDS.

The complete statistical results are summarized in Table 3, Supplementary Table S2 and S3.

**Table 3:** Linear regression models of height SDS on LAC and glucose in GSD patients (*n* = 35)

Coefficients^a^						
Model		Unstandardized Coefficients		Standardized Coefficients	t	Sig.
		B	Std. Error	Beta		
1	(Constant)	1.241	1.249		0.993	0.329
	IGF1 SDS	0.441	0.262	0.272	1.682	0.104
	BMI SDS	− 0.043	0.171	− 0.041	− 0.249	0.806
	LAC	− 0.411	0.150	− 0.520	−2.738	0.011
	GLU	− 0.439	0.251	− 0.291	−1.745	0.092
	UA	0.001	0.002	0.064	0.285	0.778
	TG	0.065	0.198	0.059	0.328	0.745

a Dependent Variable: height SDS

## Discussion

In this cohort of 38 pediatric patients with GSD, we demonstrated that fluctuations in lactate is independent predictor of linear growth failure. Hyperlactatemia was negatively correlated with height SDS, even after adjustment for IGF1 levels. This suggests that, in GSD, acute metabolic derangements may exert a more immediate impact on growth than circulating growth factors alone. However, continuous glucose monitoring data and precise hypoglycemic episode counts were not available in our cross-sectional dataset, precluding direct evaluation of hypoglycemia’s independent effect on growth. Prospective studies incorporating systematic continuous glucose tracking are needed to determine whether hypoglycemia per se—rather other metabolic derangements—contributes to growth impairment in GSD.

Subgroup analysis revealed a distinct pattern in GSD I: here, IGF1 SDS remained the sole significant predictor of height when bootstrapped confidence intervals were considered. This suggest an endocrine component to the growth impairment seen in GSD I-consistent with prior reports of GH insensitivity and reduced GH secretion in these patients-while reinforcing that tight metabolic control retains paramount importance across all GSD types [12, 17]. And Our data reinforce the clinical relevance of monitoring and potentially treating functional IGF1 deficiency—either through nutritional optimization or targeted endocrine therapies—in this subgroup.

The lack of a significant association between BMI SDS and height SDS in our cohort suggests that overall nutritional status may not fully capture the complex interplay between substrate availability, hormonal balance, and growth. While adequate caloric intake and cornstarch therapy remain essential in GSD management, our findings highlight the need for more nuanced metabolic monitoring, including regular assessment of lactate and glucose patterns, to guide therapy adjustments.

Literature on the impact of standard treatment and treatment adherence on growth outcomes in GSD is mixed. For instance, a retrospective cohort of 113 Chinese GSD Ib patients reported no significant improvement in the difference between actual height and target height (Δ height z score) after uncooked cornstarch therapy [18]. In contrast, an Italian cohort of 30 patients (GSD 0a, VI, and IXa–c) who began individualized dietary regimens early experienced median increases of + 0.6 height SDS and + 0.5 weight SDS [19]. In both GSD I and GSD III groups, implementation of continuous glucose monitoring to fine-tune cornstarch dosing led to significant gains in linear growth [14]. Furthermore, a recent multicenter international Glyde study of Glycosade^®^ (extended-release waxy maize cornstarch) further underscores the far‐reaching impact of treatment adherence on clinical outcomes [20]. In that randomized, double‐blind crossover trial, Glycosade^®^ significantly prolonged fasting duration in GSD I patients and extended ketosis‐free intervals in ketotic subtypes (GSD III/VI/IX) compared with uncooked cornstarch. Notably, 69% of participants chose to continue Glycosade^®^ long term at the end of the blinded crossover phase, and during the 2-year chronic follow-up, they maintained stable metabolic control markers while requiring significantly fewer daily doses. However, whether enhanced adherence translated to improved height outcome remained to be explored. Although questionnaire data indicated that all patients in our cohort adhered to cornstarch regimens, the lack of standardized, prospective adherence metrics limits our ability to evaluate the true consistency of treatment over time. Together, these findings imply that sustained optimization of glucose and substrate levels—not merely caloric adequacy—can ameliorate growth deficits, though prospective, controlled trials are still needed to confirm causality and delineate the magnitude of benefit.

Genotype may further modulate growth outcomes. In GSD I, individuals homozygous for severe *G6PC* mutations often exhibit more pronounced clinical severity [21], but height-specific genotype–phenotype correlations have not been well characterized. In our cohort, most patients were diagnosed in other centers, so we did not have consistent access to detailed molecular analyses and were unable to perform genotype–phenotype evaluations regarding growth. Larger studies assessing residual enzyme activity alongside growth trajectories could inform personalized prognosis and management.

Several limitations should be considered. First, the cross-sectional design and modest sample size, particularly within subgroups, may limit the generalizability of our findings and the stability of coefficient estimates. Second, our reliance on single-time-point biochemical measurements may not fully capture the chronicity or variability of metabolic disturbances. Continuous monitoring of glucose and lactate levels could provide a more comprehensive assessment of the metabolic state. Third, functional studies and external validation are needed to confirm the observed associations. Finally, although we excluded patients with overt endocrine or renal comorbidities, subclinical hormonal dysfunction (e.g., abnormalities in the growth hormone axis) was not systematically evaluated and may have confounded the associations observed.

Future research should explore prospective interventions aimed at improving both metabolic and endocrine parameters. For example, early initiation of therapies to reduce lactate accumulation could be evaluated for their impact on growth trajectories. Likewise, randomized trials assessing growth hormone therapy in carefully selected GSD I patients could provide clarity on the therapeutic potential of endocrine augmentation. Integrating continuous metabolic monitoring with periodic endocrine evaluations may facilitate personalized treatment plans that optimize growth and reduce long-term morbidity.

## Conclusions

In pediatric GSD, growth impairment is closely associated with metabolic dysregulation, with hyperlactatemia serving as key predictor of reduced height SDS in the overall cohort. Specifically, in GSD I, reduced IGF1 levels emerges as a stable predictor of short stature. These insights advocate for a dual focus on metabolic control and endocrine health in the clinical management of GSD and provide a rationale for future interventional studies targeting both pathways.

## Supplementary Information

Supplementary Material 1

Supplementary Material 2

Supplementary Material 3

## Data Availability

The data that support the findings of this study are available from the corresponding author upon reasonable request.

## Abbreviations

GSD: Glycogen Storage Disease
IGF1: Insulin-like Growth Factor 1
BMI: Body Mass Index
SDS: Standard Deviation Scores
ALT: Alanine Aminotransferase
AST: Aspartate Aminotransferase
UA: Uric Acid
TC: Total Cholesterol
TG: Triglycerides
GLU: Glucose
SPSS: Statistical Package for the Social Sciences
IRB: Institutional Review Board
