# Supplementary material for: Growth impairment in glycogen storage disease type I versus types III/VI/IX: a cross-sectional study
Source: BMC Pediatr. 2025 Oct 6;25:773. doi: 10.1186/s12887-025-06053-1 (PMC12502152; doi:10.1186/s12887-025-06053-1)
Supplement: Supplementary file 1 — Supplementary Material 1 [file 12887_2025_6053_MOESM1_ESM.docx]

Table 1 Summary of genetic findings with glycogen storage disorder

| Patient ID | GSD type | Variants | Protein | Coding impact | Diagnosis Confirmation |
| --- | --- | --- | --- | --- | --- |
| P01 | Ia | NA | NA | NA | genetical and clinical |
| P02 | Ib | c.446G>A | p.Gly149Glu | missense | genetical and clinical |
| P03 | IX | NA | NA | NA | genetical and clinical |
| P04 | Ia | NA | NA | NA | genetical and clinical |
| P05 | Ib | c.446G>A | p.Gly149Glu | missense | genetical and clinical |
| P06 | VI | c.1969+1G>A | NA | splice | genetical and clinical |
| P07 | VI | c.698G>A；c.2467C>T | p.Gln823X | nonsense | genetical and clinical |
| P08 | III | NA | NA | NA | genetical and clinical |
| P09 | IX | NA | NA | NA | genetical and clinical |
| P10 | IX | c.884G>A | p.Arg295His | missense | genetical and clinical |
| P11 | IX | NA | NA | NA | genetical and clinical |
| P12 | Ia | c.326G>A; c.648G>T | (p.Cys109Tyr)(p.Leu216=) | NA | genetical and clinical |
| P13 | Ib | NA | NA | NA | genetical and clinical |
| P14 | Ib | c.446G>A | p.Gly149Glu | missense | genetical and clinical |
| P15 | Ib | c.343G>A | p.Gly115Arg | missense | genetical and clinical |
| P16 | VI | NA | NA | NA | genetical and clinical |
| P17 | Ib | c.446G>A,c.572C>T | p.P191L | NA | genetical and clinical |
| P18 | Ib | c.446G>A | p.Gly149Glu | missense | genetical and clinical |
| P19 | Ia | c.648G>T | p.Leu216= | NA | genetical and clinical |
| P20 | Ib | c.446G>A | p.Gly149Glu | missense | genetical and clinical |
| P21 | Ib | NA | NA | NA | genetical and clinical |
| P22 | IX | c.884G>A | p.Arg295His | missense | genetical and clinical |
| P23 | Ia | NA | NA | NA | genetical and clinical |
| P24 | Ib | c.1042_1043del CT | p.Leu348Valfs*53 | Frameshift, nonsense | genetical and clinical |
| P25 | IX | c.3210_3212del | p.Arg1070del | Small deletion | genetical and clinical |
| P26 | Ia | c.648G>T; c.1022T>A | p.Leu216Leu; p.I1e341Asn |  | genetical and clinical |
| P27 | Ia | NA | NA | NA | genetical and clinical |
| P28 | Ia | NA | NA | NA | genetical and clinical |
| P29 | Ia | c.262delG | p.Val88fs | frameshift | genetical and clinical |
| P30 | Ia | c.648G>T; c.1022T>A | p.Leu216Leu; p.I1e341Asn |  | genetical and clinical |
| P31 | Ia | NA | NA | NA | genetical and clinical |
| P32 | Ia | c.648G>T | p.Leu216Leu | synonymous | genetical and clinical |
| P33 | VI | c.2467C>T | p.Gln823X | nonsense | genetical and clinical |
| P34 | VI | NA | NA | NA | genetical and clinical |
| P35 | III | c.1735+1G>T; c.1932_1934delTAC | NA | splice | genetical and clinical |
| P36 | Ib | NA | NA | NA | genetical and clinical |
| P37 | Ia | NA | NA | NA | genetical and clinical |
| P38 | VI | c.2467C>T | p.Gln823X | nonsense | genetical and clinical |
