# Supplementary material for: Growth impairment in glycogen storage disease type I versus types III/VI/IX: a cross-sectional study
Source: BMC Pediatr. 2025 Oct 6;25:773. doi: 10.1186/s12887-025-06053-1 (PMC12502152; doi:10.1186/s12887-025-06053-1)
Supplement: Supplementary file 2 — Supplementary Material 2 [file 12887_2025_6053_MOESM2_ESM.docx]

**Table S2.** LASSO Regression Coefficients for Predicting Height SDS in GSD I Patients

| **Predictor** | **Coefficient** |
| --- | --- |
| (Intercept) | –2.3004167 |
| LAC | –0.6818860 |
| IGF1 SDS | 0.5241917 |
| GlU | –0.6246372 |
