# Supplementary material for: Growth impairment in glycogen storage disease type I versus types III/VI/IX: a cross-sectional study
Source: BMC Pediatr. 2025 Oct 6;25:773. doi: 10.1186/s12887-025-06053-1 (PMC12502152; doi:10.1186/s12887-025-06053-1)
Supplement: Supplementary file 3 — Supplementary Material 3 [file 12887_2025_6053_MOESM3_ESM.docx]

**Table S2.1** Bootstrap Coefficient Estimates for LASSO Model Predicting Height SDS in GSD I Patients

| **Predictor** | **Mean** | **95% CI Lower** | **95% CI Upper** |
| --- | --- | --- | --- |
| LAC | –0.6963499 | –1.6739510 | 0.0052865 |
| IGF1SDS | 0.5625413 | 0.1030077 | 1.1656068 |
| Glucose | –0.5638938 | –1.2791075 | 0.1170184 |

Mean estimates and 95% confidence intervals for each predictor.

**Table S2.2**. Model Mean Squared Error (MSE) for GSD Patients

| **Metric** | **Mean** | **95% CI Lower** | **95% CI Upper** |
| --- | --- | --- | --- |
| MSE | 1.9361 | 1.5424 | 3.6281 |

Bootstrap mean MSE and its 95% confidence interval for the LASSO model.
